# Knockdown of KDM1A suppresses tumour migration and invasion by epigenetically regulating the TIMP1/MMP9 pathway in papillary thyroid cancer

**DOI:** 10.1111/jcmm.14311

**Published:** 2019-06-18

**Authors:** WenQian Zhang, Wei Sun, Yuan Qin, CangHao Wu, Liang He, Ting Zhang, Liang Shao, Hao Zhang, Ping Zhang

**Affiliations:** ^1^ Department of Thyroid Surgery The First Hospital of China Medical University Shenyang Liaoning Province China

**Keywords:** H3K4me2, KDM1A, lymphatic metastasis, thyroid cancer, TIMP1

## Abstract

Epigenetic dysregulation plays an important role in cancer. Histone demethylation is a well‐known mechanism of epigenetic regulation that promotes or inhibits tumourigenesis in various malignant tumours. However, the pathogenic role of histone demethylation modifiers in papillary thyroid cancer (PTC), which has a high incidence of early lymphatic metastasis, is largely unknown. Here, we detected the expression of common histone demethylation modifiers and found that the histone H3 lysine 4 (H3K4) and H3 lysine 9 (H3K9) demethylase KDM1A (or lysine demethylase 1A) is frequently overexpressed in PTC tissues and cell lines. High KDM1A expression correlated positively with age <55 years and lymph node metastasis in patients with PTC. Moreover, KDM1A was required for PTC cell migration and invasion. KDM1A knockdown inhibited the migration and invasive abilities of PTC cells both in vitro and in vivo. We also identified tissue inhibitor of metalloproteinase 1 (TIMP1) as a key KDM1A target gene. KDM1A activated matrix metalloproteinase 9 (MMP9) through epigenetic repression of TIMP1 expression by demethylating H3K4me2 at the TIMP1 promoter region. Rescue experiments clarified these findings. Altogether, we have uncovered a new mechanism of KDM1A repression of TIMP1 in PTC and suggest that KDM1A may be a promising therapeutic target in PTC.

## 1 INTRODUCTION

Thyroid cancer is the most common endocrine malignancy and the fifth most frequent cancer in women. It accounted for 56 870 newly diagnosed malignancies and 2010 deaths in the United States in 2017.^1^ Thyroid cancer has been classified into four subtypes; Papillary thyroid cancer (PTC) is the main type, representing approximately 85%‐90% of all cases and PTC incidence has steadily increased over the past decades.^2^ Although it usually has a favourable long‐term survival rate and most patients can be treated successfully with surgery and radioactive iodine (RAI), the prognosis may be unpredictable for patients with lymph node metastasis.^3^ Accordingly, there is a need for better understanding of the molecular mechanism of PTC metastatic behaviour.

As a key type of histone modification, methylation plays a crucial role in epigenetic regulation of gene expression. Histone methylation mainly acts on lysine residues and regulates either the repression or activation of gene expression. Histone lysine methylation is reversibly modulated by specific lysine methyltransferases (KMTs) and lysine demethylases (KDMs) and is involved in various biological processes and diseases in humans.^4^, ^5^, ^6^, ^7^ As the first demethylase discovered, KDM1A is the best characterized KDM. KDM1A is also known as LSD1 and AOF2,^8^ and can interact with various protein complexes (eg CoREST, NuRD, RCOR2), receptors (eg oestrogen, androgen, TLX), non‐histone proteins (eg p53, E2F1, DNMT1), and transcription factors (eg TLA and SNAIL).^9^ KDM1A promotes carcinogenesis progression by removing the methyl groups from methylated histone H3 lysine 4 (H3K4) and H3 lysine 9 (H3K9). Recent studies have shown that KDM1A is overexpressed in multiple malignant tumours, including lung cancer, cervical cancer, oesophageal cancer and ovarian cancer. Elevated KDM1A levels are also associated with tumour stage, histological grade and lymph node metastasis.^10^, ^11^, ^12^, ^13^ Furthermore, these studies have suggested that KDM1A might function as an oncogene by regulating the degree of histone methylation of its target gene.

Tissue inhibitors of metalloproteinases (TIMPs) comprise a four‐member family: TIMP1, TIMP2, TIMP3 and TIMP4, which bind matrix metallopeptidases (MMPs) in a 1:1 ratio to inhibit their proteolytic activity.^14^ These endogenous secreted proteins inhibit all MMPs, and each TIMP targets multiple enzymes. TIMP1 was discovered in the 1980s, and contains 184‐194 amino acids and has a molecular weight of 21 kDa. As an important regulator of MMP9, it is functionally relevant, as demonstrated by the association of the compromised TIMP1/MMP9 ratio in prostate cancer and liver cancer.^15^, ^16^ However, the function of TIMP1 in PTC is unknown.

Compared with other epigenetic modifications such as DNA methylation and non‐coding RNA abnormality, little is known about the role of histone demethylation in PTC. Here, we investigated which KDMs are abnormally expressed in PTC tissues. We found that KDM1A was overexpressed in PTC tissues compared to the adjacent non‐cancerous tissues. We then evaluated the relationship between KDM1A and the clinical features of PTC, and detected the migration and invasive ability of PTC cells after KDM1A down‐regulation. We report, for the first time, that TIMP1 is a target gene of KDM1A and that KDM1A may regulate TIMP1 via its demethylase activity. Our results could partly clarify the molecular mechanisms of PTC metastasis.

## 2 MATERIALS AND METHODS

### 2.1 Patient samples and tissue microarray

We obtained fresh tumour tissue and the corresponding non‐cancerous tissue from 60 patients with PTC. After resection, the samples were snap‐frozen in liquid nitrogen and stored at −80°C until RNA and protein extraction. For the tissue microarray (TMA), we obtained 61 paired samples of PTC tissue and adjacent non‐cancerous tissue and 94 samples of PTC tissue alone from 155 patients. All patients had undergone surgical treatment at The First Affiliated Hospital of China Medical University, Shenyang, China, between September 2014 and February 2017 and had not received preoperative treatment. All specimens were confirmed by histopathology. Written informed consent forms were issued and signed by all patients. We obtained approval from The First Affiliated Hospital of China Medical University Ethics Committee. The described clinicopathological characteristics included age, gender, tumour size, lymph node metastasis, extrathyroidal extension, and tumour‐node‐metastasis stage. Tumours were staged based on criteria specified in the eighth edition of the American Joint Committee on Cancer on Cancer Staging Manual.

### 2.2 Total RNA extraction and quantitative reverse transcription–PCR

Total RNA from frozen tissue specimens and cultured cells were extracted using RNAiso Plus (Takara, Dalian, China) and quantified using a NanoDrop 1000 spectrophotometer (Thermo Scientific, Wilmington, DE, USA). RNA (2 µg) was reverse‐transcribed into single‐stranded complementary DNA using an RT kit (RR036A, Takara). Quantitative reverse transcription–PCR (qRT‐PCR) was performed using SYBR Premix Ex Taq II (Takara) on a LightCycler 480 system (Roche, Indianapolis, IN). Each sample was run in triplicate and normalized to glyceraldehyde‐3‐phosphate dehydrogenase (GAPDH). The relative fold change was the fold change compared with GAPDH.

### 2.3 Cell culture

The human PTC cell line BCPAP was obtained from DSMZ (Braunschweig, Germany). The human normal thyroid follicular epithelial cell line Nthy‐ori 3‐1 and the human PTC cell line K1 were purchased from the European Collection of Authenticated Cell Culture (ECACC, Salisbury, UK). The human PTC cell line IHH‐4 was obtained from the Health Science Research Resources Bank (Osaka, Japan), and the TPC1 cell line was a gift from Professor Meiping Shen (Department of General Surgery, The First Affiliated Hospital of Nanjing Medical University, Nanjing, Jiangsu). The Nthy‐ori 3‐1 cells were cultured in Roswell Park Memorial Institute (RPMI) 1640 medium containing 2 mmol/L glutamine and 10% foetal bovine serum (FBS). The BCPAP cells were maintained in RPMI 1640 medium with 10% FBS. The TPC1 and K1 cells were cultured in high‐glucose Dulbecco's modified Eagle's medium (DMEM) supplemented with 10% FBS. IHH‐4 cells were maintained in a 1:1 mixture of RPMI 1640 and high‐glucose DMEM supplemented with 10% FBS. All cells were cultured at 37°C in a humidified atmosphere with 5% CO2.

### 2.4 RNA interference and transfection protocol

The short interfering RNAs (siRNAs) against KDM1A (si‐KDM1A) and TIMP1 (si‐TIMP1) were synthesized by GenePharma (Suzhou, China). The siRNA sequences were as follows: si‐KDM1A (1), sense, 5′‐CCGGAUGACUUCUCAAGAATT‐3′; antisense, 5′‐UUCUUGAGAAGUCAUCCGGTT‐3′. si‐KDM1A (2), sense, 5′‐GCCACCCAGAGAUAUUACUTT‐3′, antisense 5′‐AGUAAUAUCUUGGGUGG CTT‐3′. si‐TIMP1, sense, 5′‐UUCUCCGAACGUGUCACGUTT‐3′; antisense, 5′‐UAUAAGGUGGUCUGGUUGATT‐3. Negative control (NC), sense, 5′‐UUCUCCGAACGUGUCACGUTT‐3′. The shRNA against KDM1A were synthesized by GenePharma (Suzhou, China), the sequences were as follows: forward oligo:GATCCGGCAAAGAAGCATCTGAAGTAAAGGTACCTTTACTTCAGATGCTTCTTTGTTTTTG and Reverse oligo: AATTCAAAAACAAAGAAGCATCTG AAGTAAAGGTACCTTTACTTCAGATGCTTCTTTGCCG. TPC1 and IHH‐4 cells were transfected using Lipofectamine 2000 (Invitrogen, CA) according to the manufacturer's protocol. Following 48‐72‐hours incubation, the cells were harvested for mRNA or protein analysis.

### 2.5 Immunohistochemistry

TMA sections were baked at 65°C for 30 min, then deparaffinized and rehydrated with different concentrations of xylene and alcohol gradients, respectively. Endogenous peroxidase activity was blocked with 3% H_2_O_2_. Then, the sections were treated with citrate buffer and microwave‐heated for 20 min before overnight incubation at 4°C with anti‐KDM1A antibody (1:200; CST, MA). The immunohistochemical staining score was evaluated using the semi‐quantitative Remmele scoring system.^17^ Briefly, the staining intensity was scored as 0 (no staining), 1 (weak staining = light yellow), 2 (moderate staining = yellow brown) or 3 (strong staining = brown). The proportion of positive PTC cells was scored as 0 (no positive tumour cells), 1 (<10% positive tumour cells), 2 (10‐50% positive tumour cells) or 3 (>50% positive tumour cells). The staining index (SI) was calculated by multiplying the scores for staining intensity and proportion. Positive KDM1A expression was defined as SI ≥ 3.

### 2.6 Wound healing assay

PTC cells were cultured in six‐well plates and incubated for 24‐48 hours until approximately 85%‐90% confluent. After transfection with si‐KDM1A (1), si‐KDM1A (2) and si‐NC, wounds were created in the cell cultures using a 200 μL pipette tip. Floating cells were washed away with phosphate‐buffered saline. The cultures were then incubated in serum‐free medium for 24 hours. Then, the distances migrated by the cells were imaged under a microscope, with three observation fields per well. The percentage of would closure was measured using Image J (NIH, MD, USA) software.

### 2.7 Transwell assay

After 24 h transfection, we performed invasion and migration assays using Transwell chambers (Millipore, Bedford, MA) with or without Matrigel (BD, Bedford, MA). PTC cells (migration: 1.5 × 10^4^ TPC1 cells and 3 × 10^4^ IHH‐4 cells; invasion: 3 × 10^4^ TPC1 cells and 5 × 10^4^ IHH‐4 cells) in serum‐free medium were added to the upper chamber, and the lower chamber was filled with 500 μL medium supplemented with 10% FBS. Following 24 hours incubation, the cells were fixed with 4% formaldehyde and stained with 0.1% crystal violet. The cells on the underside of the membrane were counted using Image J software. The experiments were carried out independently in triplicate.

### 2.8 Western blotting

Total proteins were extracted using a Total Protein Extraction Kit (KeyGEN, Nanjing, China) and quantified using a bicinchoninic acid (BCA）protein assay kit (Beyotime, Shanghai, China). Equal amounts of proteins (20‐30 µg) were separated by 10% sodium dodecyl sulphate–polyacrylamide gel electrophoresis, and then electrotransferred onto 0.45 or 0.2 µm polyvinylidene difluoride membranes (Millipore). After blocking with 5% skim milk, the membranes were incubated overnight at 4°C with primary antibodies (1:1000) and at room temperature for 2 hours with secondary antibodies (1:5000). Protein bands were detected by chemiluminescence (Thermo, MA, USA). The following primary antibodies were used: KDM1A (CST, #2184), TIMP1 (abcam, Shanghai, China, ab109125), MMP9 (abcam, ab76003), MMP2 (abcam, ab37150), H3K4me2 (abcam, ab32356), H3K9me2 (abcam, ab176882), GAPDH (Sigma‐Aldrich, MO, USA #G9545) and total histone H3 (CST, #4499).

### 2.9 Chromatin immunoprecipitation

Chromatin immunoprecipitation (ChIP) assays were performed as indicated in an enzymatic ChIP kit (CST, #9002). Briefly, 3 × 10^7^ IHH‐4 cells or 1.5 × 10^7^ TPC1 cells were crosslinked using 1% formaldehyde. Micrococcal nuclease (0.5 µL) was added per IP and sonicated into 200‐500 bp fragments. Approximately 5 μg antibody was added per IP and incubated overnight at 4°C. Rabbit immunoglobulin G (IgG）was used as a control. Protein G agarose beads were added and incubated at 4°C for 2 hours, and then the chromatin and antibodies were eluted at 65°C for 4 hours in IP elution buffer. The cross‐links were reversed, and the DNA was purified using a spin column and quantified by qRT‐PCR. The primers for the TIMP1 promoter and upstream regions were as follows: TIMP1 (+1051) forward, 5′‐AAAGCTGGTGGGCAAGGATT‐3′; reverse, 5′‐CCGGGATTCAAACAGAGGCT‐3′. TIMP1 (+1626): forward, 5′‐ATCTCCCTCCACTGCTGCTA‐3′; reverse, 5′‐ACATCCCCCAAGCTCCCTAT‐3′. TIMP1 (+1868): forward, 5′‐GGCGGCTTGGAAGGAATAGT‐3′; reverse, 5′‐TAAATGTCCAGCCTAGGGGC‐3′.

### 2.10 Gelatin zymography

The cells were cultured in high‐glucose DMEM supplemented with 10% FBS until approximately 70%‐80% confluent, washed twice, and the culture was continued in serum‐free DMEM for 24 hours. The conditioned medium was collected and centrifuged. The conditioned media from all samples were adjusted to contain the same protein concentrations. An 8% acrylamide gel containing gelatin was prepared. After electrophoresis, the gel was incubated in incubation buffer for 24 hours at 37°C. The gel was stained with 0.5% Coomassie brilliant blue R‐250 and photographed after elution.

### 2.11 Metastasis assay in nude mice

Briefly, 1 × 10^7^ TPC1 cells of sh‐KDM1A transfection and sh‐NC transfection were injected intravenously through the tail vein into 4‐5‐week‐old severe combined immunodeficient nude mice. After 6 weeks, the number of tumour nodules formed on the lung surfaces was counted.

### 2.12 Statistical analysis

All statistical analyses were performed using SPSS 22.0 (IBM, Chicago, IL). The correlation between KDM1A expression and the patients’ clinical characteristics were examined using Pearson's chi‐square test. The correlation between KDM1A and TIMP1 expression were evaluated using Pearson correlation analysis. Student's *t* test was used to analyse the comparison of cell migration and invasion, qRT‐PCR, and ChIP experiments. A two‐sided test was considered statistically significant at *P* < 0.05.

## 3 RESULTS

### 3.1 KDM1A expression was elevated in PTC and correlated with lymph node metastasis

**Figure 1:**
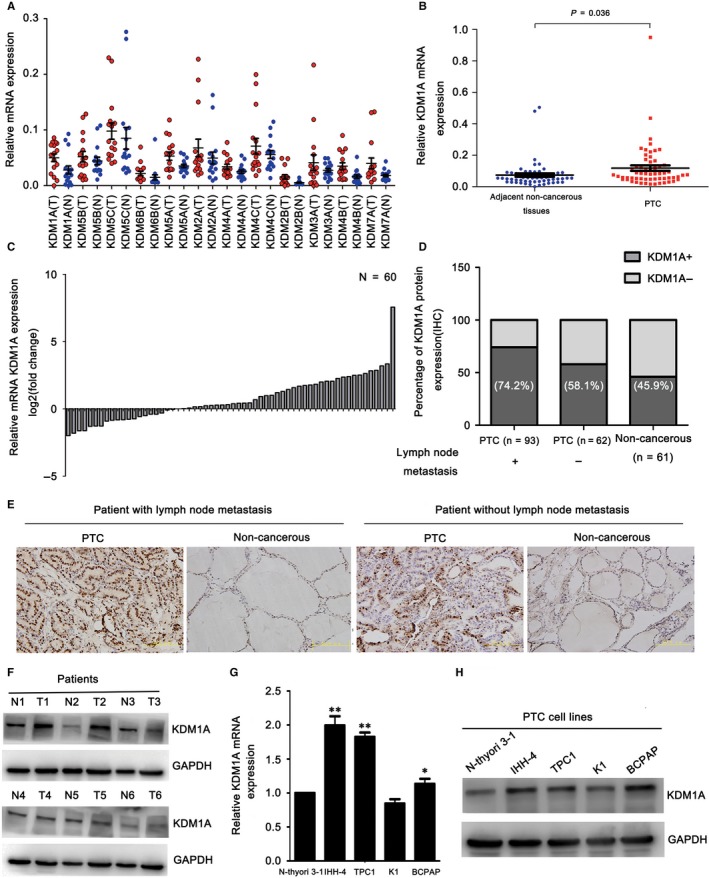
KDM1A was overexpressed in papillary thyroid cancer (PTC) tissues and cell lines. (A) Histone demethylase mRNA expression levels in 16 pairs of PTC and adjacent non‐cancerous tissues. (B) Relative KDM1A mRNA expression levels in 60 pairs of PTC and adjacent non‐cancerous tissues. (C) Fold change of KDM1A mRNA expression in PTC and corresponding adjacent non‐cancerous tissues. (D) KDM1A was expressed high in PTC and positive expression of KDM1A correlated with lymph node metastasis. (E) Representative photographs from IHC analysis of KDM1A protein levels in normal and tumour samples with or without lymph node metastasis. Scale bars: 50 μm. (F) Western blot analysis of relative KDM1A protein levels in a small sample of PTC (T) and corresponding adjacent non‐cancerous tissues (N). (G‐I) Analysis of relative KDM1A mRNA and protein levels in the Nthy‐ori 3‐1 cell line and four human PTC cell lines (IHH‐4, TPC1, K1, BCPAP) by qRT‐PCR (G) and western blotting (H), respectively. GAPDH was used as an internal loading control. *P < 0.05; **P < 0.01

Initially, to identify histone demethylation modifiers with oncogenic properties in PTC, we assessed the histone demethylation modifiers that may be highly expressed in 16 pairs of PTC tissue and the adjacent non‐cancerous tissue using qRT‐PCR. KDM1A, KDM5A and KDM7A were up‐regulated in the PTC tissues as compared to the non‐cancerous tissue (Figure 1A). Then, we expanded the sample size to 60 pairs of PTC tissue and non‐cancerous tissue, and found no difference in KDM5A expression between the paired tissues. RNA interference indicated that KDM7A may not be important for migration and invasion in PTC. These results led to our selection of KDM1A as a primary candidate for subsequent functional analyses.

PTC tissues had increased KDM1A mRNA expression compared to the paired adjacent non‐cancerous tissues (Figure 1B,C). KDM1A protein expression levels were detected from TMA via immunohistochemistry (IHC). As shown in (Figure 1D,E), PTC tissues had significantly elevated KDM1A protein levels (Table 1), especially in tissue from patients with lymph node metastasis. The IHC score was used to determine whether KDM1A expression level was associated with the clinicopathological features of the patients with PTC. As shown in (Table 2), KDM1A positive expression was significantly related to age <55 years (*P* = 0.019) and lymph node metastasis (*P* = 0.035). The high KDM1A expression in PTC tissues was confirmed by western blotting using a small amount of fresh tissues (Figure 1F). As the PTC tissue had higher KDM1A expression than the non‐cancerous tissue at both mRNA and protein level, we assessed whether PTC cell lines had up‐regulated KDM1A expression. qRT‐PCR and western blotting showed that the IHH‐4, TPC1 and BCPAP PTC cells expressed higher levels of KDM1A, whereas its expression was lower in the K1 PTC cell line than in the Nthy‐ori 3‐1 human normal thyroid follicular epithelial cell line (Figure 1G,H). Taken together, these findings suggest that KDM1A may play an oncogenic role in PTC development.

**Table 1:** Immunohistochemistry analysis of KDM1A protein levels in 61 paired papillary thyroid cancer (PTC) tissues and adjacent non‐cancerous tissues

Sample	KDM1A		*P*
	+	−	
Non‐cancerous tissues	28 (46%)	33 (54%)	0.003
PTC tissues	105 (68%)	50 (32%)	

**Table 2:** Correlation between KDM1A expression and clinicopathological features in patients with papillary thyroid cancer (n = 155)

Characteristic	n	KDM1A		*P*
		+	−	
Age, y				
＜55	120	87	33	0.019
≥55	35	18	17	
Gender				
Male	41	31	10	0.209
Female	114	74	40	
Extrathyroidal invasion				
Yes	50	35	15	0.678
No	105	70	35	
Lymph‐node metastasis				
Yes	93	69	24	0.035
No	62	36	26	
Tumour size				
<2 cm	35	22	13	0.482
≥2 cm	120	83	37	
Tumour‐node‐metastasis staging				
Ⅰ‐Ⅱ	145	99	46	0.588
Ⅲ‐Ⅳ	10	6	4	

### 3.2 KDM1A knockdown inhibited PTC cell migration and invasion

**Figure 2:**
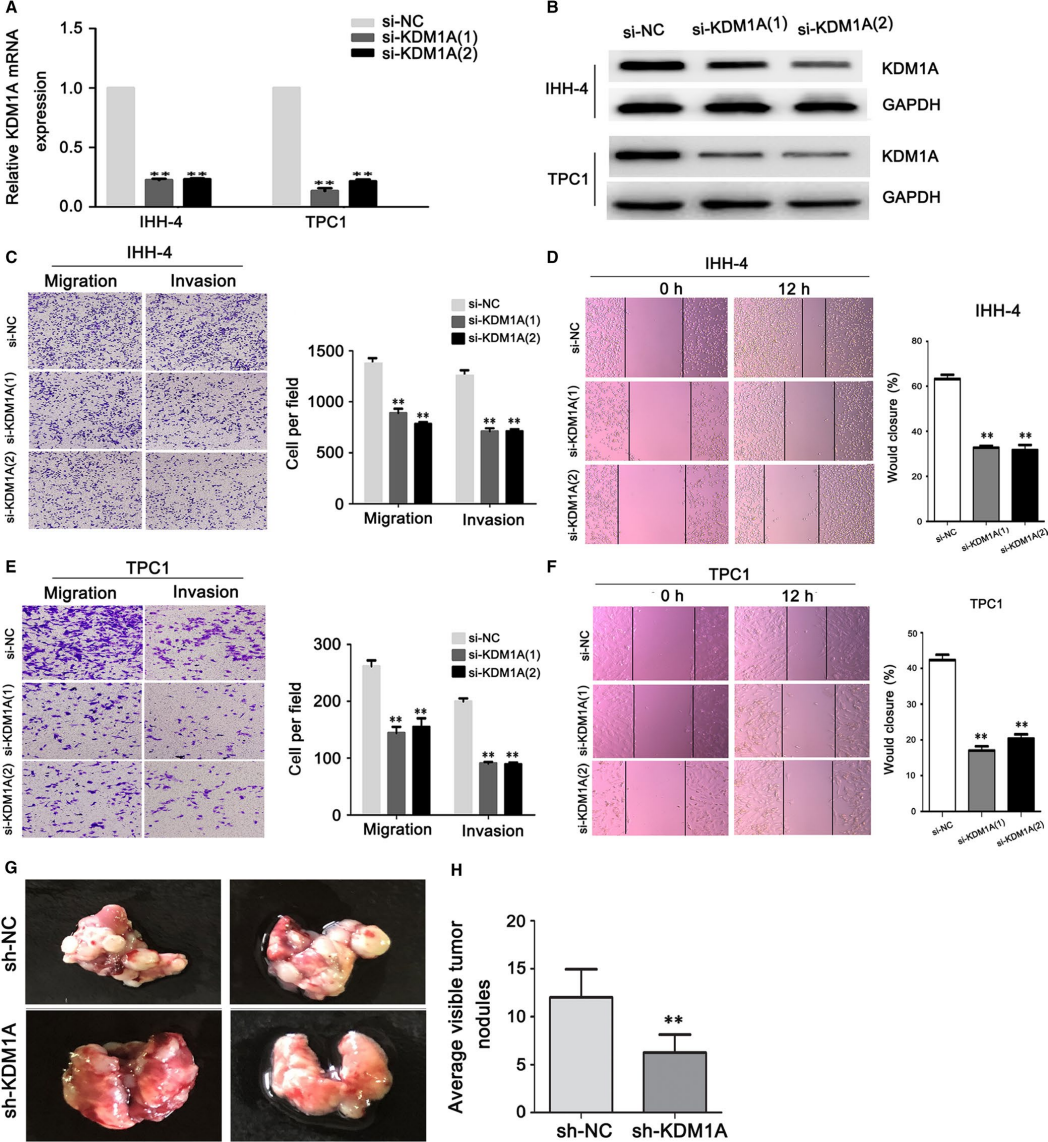
KDM1A knockdown inhibited papillary thyroid cancer (PTC) cell migration and invasion. (A and B) Analysis of KDM1A interference efficiency after si‐KDM1A and si‐NC transfection in PTC cells by PCR (A) and western blotting (B). (C‐F) Transwell and wound healing assay evaluation of cell migration and invasion after si‐KDM1A or si‐NC transfection in IHH‐4 (C and D) and TPC1 cells (E and F). (G and H) Representative lung metastasis nodules from node mice were shown. The formation of metastatic nodules at the lung surface could be significantly suppressed in sh‐KDM1A group *P < 0.05; **P < 0.01

To further understand the function of KDM1A in PTC, we knocked down KDM1A in the IHH‐4 and TPC1 KDM1A‐overexpressing cell lines using two siRNA sequences, both of which could down‐regulate KDM1A effectively (Figure 2A,B). In Section 3.1, we reported that positive KDM1A expression in PTC tissues correlated significantly with lymph node metastasis. This result suggests that KDM1A might promote PTC cell migration and invasion. To assess the effect of KDM1A on the migration and invasive ability of PTC cells, we performed transwell and wound healing assays using the IHH‐4 and TPC1 cell lines. KDM1A knockdown inhibited the migration and invasive ability of IHH‐4 and TPC1 cells remarkably (Figure 2C,E), and the wound healing assay confirmed the effect of KDM1A depletion on PTC cell migration capability (Figure 2D,F). To further investigate the effect of KDM1A on tumour metastasis, we performed pulmonary metastasis assay in nude mice by injecting sh‐KDM1A and sh‐NC transfected PTC cells through tail vein. The result showed that the number of visible nodules in the sh‐KDM1A group were significantly increased when compared to those in the sh‐NC group (Figure 2G,H). Theses results suggested that knowdown KDM1A could inhibit PTC cell migration and invasion both in vitro and in vivo.

### 3.3 The TIMP1 gene is a key target gene of KDM1A

**Figure 3:**
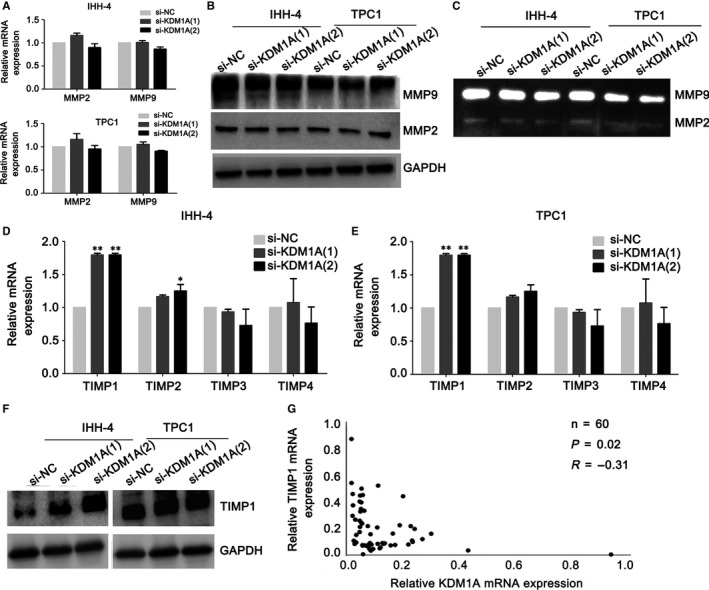
KDM1A knockdown highly up‐regulated TIMP1. (A and B) Detection of MMP9 and MMP2 levels by PCR (A) and western blotting (B) after si‐KDM1A or si‐NC transfection in IHH‐4 and TPC1 cells. (C) Gelatin zymography analysis of MMP9 activity in IHH‐4 and TPC1 cells after KDM1A knockdown. (D and E) qRT‐PCR analysis of TIMP1, TIMP2, TIMP3 and TIMP4 expression levels in IHH‐4 (D) and TPC1 cells (E) after KDM1A knockdown. (F) Western blot analysis of the effect of KDM1A knockdown on TIMP1 protein levels in IHH‐4 and TPC1 cells. GAPDH was used as an internal loading control. (G) Correlation analysis between KDM1A and TIMP1 expressions in 60 papillary thyroid cancer and tissues. *P < 0.05; **P < 0.01

To further understand the mechanisms of KDM1A promotion of metastasis in PTC, we detected the expression of MMP2 and MMP9. MMP2 and MMP9 degrade type IV collagen and play important roles in various pathophysiological processes, such as wound healing and invasiveness.^18^, ^19^ Interestingly, the si‐KDM1A groups did not have obviously altered MMP9 mRNA expression, but had significantly decreased MMP9 protein levels. In the si‐KDM1A groups, MMP2 was unaltered at both mRNA and protein level as compared with the si‐NC group (Figure 3A,B). Gelatin zymography indicated that the si‐KDM1A groups had also significantly repressed MMP9 activation compared with the si‐NC groups (Figure 3C). These results suggest that MMP9 might be regulated post‐transcriptionally, at both expression and activation level. So we speculated that TIMPs, the natural inhibitors of the MMPs, may play important roles in the KDM1A‐induced effects in PTC cells. We detected the mRNA expression of all TIMPs (ie TIMP1‐TIMP4); The results showed that TIMP1 was significantly up‐regulated in the si‐KDM1A groups in both the IHH‐4 and TPC1 cell lines (Figure 3D,E). In agreement with this finding, KDM1A knockdown also increased TIMP1 protein levels (Figure 3F). TIMPs not only form tight complexes with MMPs, they also bind to pro‐MMPs,^14^ suggesting that TIMP1 can repress both the expression and activation of MMP9. Moreover, KDM1A expression was negatively correlated with TIMP1 expression in 60 PTC tissues (Figure 3G). Therefore, we presume that TIMP1 may be a key target gene of KDM1A and that KDM1A may promote PTC cell migration and invasion by regulating TIMP1 negatively.

### 3.4 TIMP1 down‐regulation partly impaired KDM1A depletion‐mediated cell migration and invasion

**Figure 4:**
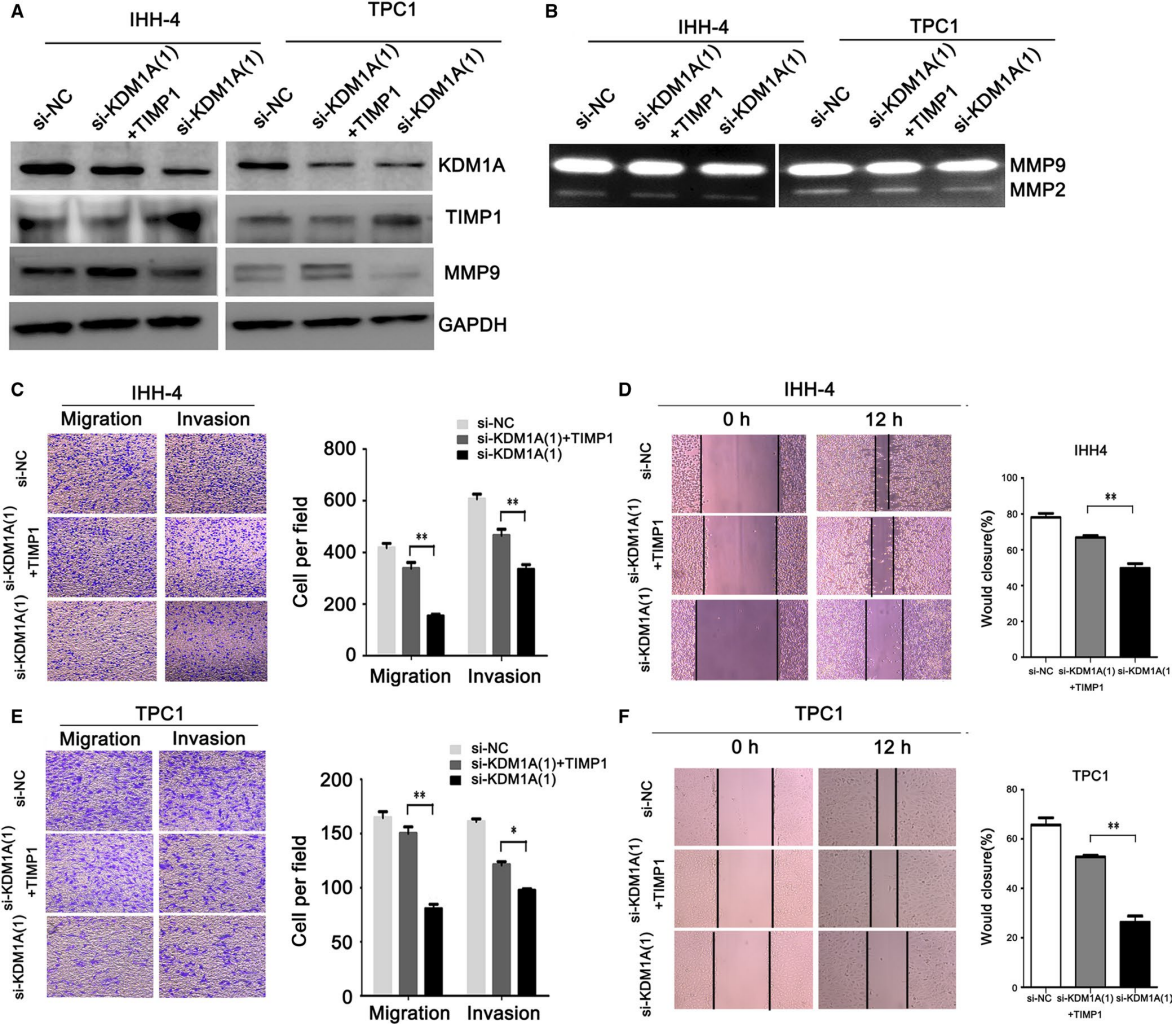
TIMP1 knockdown could rescue si‐KDM1A–induced inhibition of papillary thyroid cancer (PTC) cell migration and invasion. (A) Western blot analysis of TIMP1 levels in PTC cells following si‐KDM1A transfection, si‐KDM1A plus si‐TIMP1 co‐transfection, or si‐NC transfection. GAPDH was used as an internal loading control. (B) Gelatin zymography analysis of MMP9 activation in PTC cells following si‐KDM1A transfection, si‐KDM1A plus si‐TIMP1 co‐transfection, or si‐NC transfection. (C‐F) Transwell and Wound healing assay evaluation of the migration and invasion of IHH‐4 (C and D) and TPC1 (E and F) cells after si‐KDM1A transfection, si‐KDM1A plus si‐TIMP1 co‐transfection, or si‐NC transfection. *P < 0.05; **P < 0.01

To confirm whether TIMP1 repression is necessary for KDM1A‐induced PTC cell migration and invasion, we co‐transfected IHH‐4 and TPC1 cells with si‐KDM1A and si‐TIMP1. Western blotting showed that the co‐transfected cells had significantly decreased TIMP1 and increased MMP9 expression as compared with cells transfected with si‐KDM1A alone (Figure 4A). The co‐transfected cells also had enhanced MMP9 activation compared with cells transfected with si‐KDM1A alone (Figure 4B). We also assessed the effects of si‐KDM1A transfection, si‐KDM1A plus si‐TIMP1 co‐transfection and si‐NC transfection on cell function. TIMP1 down‐regulation could partly rescue the si‐KDM1A‐induced repression of the PTC cell migration and invasive abilities (Figure 4C‐F). However, as TIMP1 knockdown could only partly rescue si‐KDM1A‐mediated repression of the malignant behaviour in PTC cells, it indicates that TIMP1 might be just one downstream target gene regulated by KDM1A.

### 3.5 KDM1A demethylated H3K4me2 at the TIMP1 promoter

**Figure 5:**
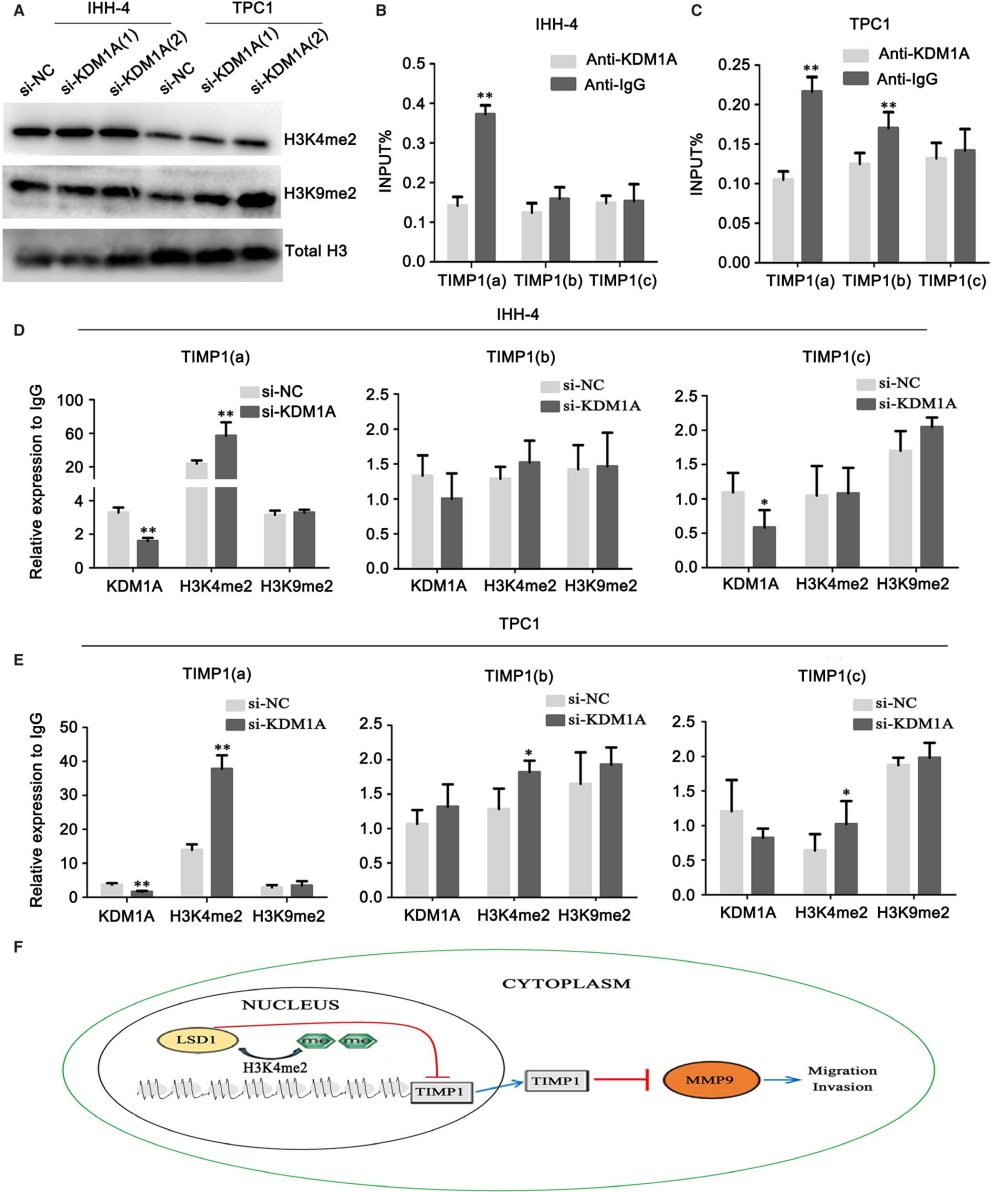
KDM1A removes H3K4me2 at the TIMP1 promoter and represses its promoter activity. (A) Western blot detection of H3K4me2 and H3K9me2 protein levels in IHH‐4 and TPC1 cells after si‐KDM1A or si‐NC transfection. Total histone H3 served as a loading control. (B and C) Analysis of levels of KDM1A occupation at TIMP1 in IHH‐4 (B) and TPC1 cells (C). Schematic drawing of the TIMP1 promoter region and ChIP‐qPCR primer set locations (a, +1051, b, +1626, c, +1868) relative to the TSS (transcriptional start site). TSS was assigned the “+1” position. Chromatin levels of KDM1A were measured by quantitative ChIP. (D and E) Analysis of the levels of occupation of KDM1A, H3K4me2, and H3K9me2 at the TIMP1 gene promoter region after si‐KDM1A or si‐NC transfection in IHH‐4 (D) and TPC1 cells (E). (F) A hypothetical representation of the regulatory pathway underlying KDM1A‐promoted cell migration and invasion. KDM1A represses the TIMP1 gene by removing the epigenetic activation marker H3K4me2. KDM1A‐mediated repression of TIMP1 results in increased MMP9 expression and activation, which are critical for cell migration and invasion. Consequently, MMP9 activation by KDM1A‐mediated repression of TIMP1 contributes to the metastasis of papillary thyroid cancer cells. *P < 0.05; **P < 0.01

The above data indicate that TIMP1 is a potential KDM1A target gene. However, the mechanism of how KDM1A regulates TIMP1 remains to be clarified. KDM1A is a transcriptional repressor because it demethylates the gene activation marker H3K4me2.^20^ To understand the mechanism of how KDM1A down‐regulation up‐regulates TIMP1 expression in PTC cells, we focused on whether KDM1A could act as a TIMP1 repressor by demethylating H3K4me2. Following si‐KDM1A transfection, global H3K4me2 and H3K9me2 levels were up‐regulated in both IHH‐4 and TPC1 cells (Figure 5A). ChIP was performed to determine whether KDM1A bound to TIMP1 and what regions KDM1A occupied. Three primers were designed at +1051(a), +1626(b), +1868(c) of the promoter region. We found that KDM1A was localized at the proximal promoter region (region a) but not the distal promoter region (region b and c) of the TIMP1 gene (Figure 5B,C). Supporting the finding of KDM1A localization at the proximal promoter region of the TIMP1 gene, KDM1A knockdown decreased KDM1A levels and increased H3K4me2 levels at the same region. Additional ChIP data demonstrated that KDM1A knockdown did not alter H3K9me2 levels at these regions (ie region a, b and c) (Figure 5D,E). These results suggest that KDM1A directly represses the TIMP1 gene by binding to its promoter and specifically demethylating H3K4me2 at the proximal promoter region. This is consistent with a previous report indicating that the KDM1A binding sites are located at the transcription start regions.^21^

## 4 DISCUSSION

The prognosis of PTC is generally favourable; however, lymph node metastases are very common: some studies have reported that lymph node metastases can be found in approximately 60% of cases.^22^ Moreover, long‐term follow‐up has shown that patients with lymph node metastases have higher recurrence rates and lower survival rates.^23^, ^24^, ^25^ In recent years, various genomic and epigenetic aberrations related to metastasis have been reported in PTC.^26^, ^27^ However, the specific mechanism of how these abnormalities affect PTC remains poorly understood.

Histone modification regulates gene expression through different ways, such as through acetylation, methylation, ubiquitination and phosphorylation. Methylation was the first histone post‐translational modifications reported in 1964.^28^ Many of these enzymes are expressed abnormally in human cancers, suggesting that they are critical regulators of cellular homeostasis. Consequently, they are potential targets for therapeutic intervention. As the first discovered histone demethylase, KDM1A is the best characterized.^29^ KDM1A belongs to the FAD (flavin adenine dinucleotide) dependent histone demethylase family and regulates gene expression as a nuclear homolog of amine oxidases, specifically in the demethylation of monomethylated and dimethylated H3K4me1/2 and H3K9me1/2. As an important regulator, KDM1A plays critical roles in physiological processes such as the regulation of embryonic development, differentiation shape and identity determination of stem and progenitor cells. It also contributes to a variety of pathological conditions, eg cancer, neuronal disorders and viral infections.^30^, ^31^ Presently, aberrant KDM1A expression is associated with metastasis and unfavourable prognosis in various cancers, suggesting a widespread oncogenic role. For example, KDM1A is overexpressed and promotes migration and invasion in prostate cancer, breast cancer, colon cancer and non–small cell lung cancer.^32^, ^33^, ^34^, ^35^, ^36^ In particular, KDM1A is overexpressed in PTC. Kong and colleagues detected KDM1A expression immunohistochemically in 57 PTC cases and 23 paracancerous tissue samples.^37^ They showed that the PTC tissues had higher KDM1A expression than the paracancerous tissues. KDM1A down‐regulation can also suppress the invasive ability of PTC cell line K1,^38^ but how KDM1A affects PTC cells remains unknown.

Here, we found that relative KDM1A mRNA overexpression in PTC tissues as compared to non‐cancerous tissues in 60 pairs of matched samples. IHC and western blotting of the protein levels confirmed this result. Moreover, our data show that KDM1A positive expression indicates a higher lymph node metastasis rate. We also detected KDM1A mRNA and protein expression in PTC cell lines. High KDM1A expression levels were observed in IHH‐4 and TPC1 cells, especially in IHH‐4 cells, which are related to cervical lymph node metastasis. Si‐KDM1A inhibited migration and invasion both in vitro and in vivo. Furthermore, we determined that the downstream metastasis‐associated MMP9 was significantly down‐regulated in the si‐KDM1A groups at protein level, but not at mRNA level. Gelatin zymography also showed that KDM1A knockdown inhibited MMP9 activation. These results are consistent with an oncogenic role of KDM1A.

It has been well‐established that MMP9 promotes invasion by being secreted outside the cell to degrade the extracellular matrix and basal membrane components such as type IV collagen. MMP9 can also activate several latent proteinases and angiogenic factors or cytokine receptors, which enhance invasion and metastasis.^39^ MMP9 is a cancer promoter in PTC, for example, MMP9 is up‐regulated and correlates with tumour diameter, lymph node metastasis, degree of PTC infiltration and clinical stage.^40^, ^41^ We found that si‐KDM1A reduced MMP9 expression and activation, suggesting that it may promote tumour progression by regulating MMP9. Intriguingly, we found that while MMP9 mRNA expression did not decrease, MMP9 protein expression was decreased significantly. This led us to speculate that MMP9 is regulated post‐transcriptionally. Accordingly, we detected the expression level of the TIMPs, which inhibit MMP proteolytic activity by binding MMPs.^42^ Our result showed that the si‐KDM1A groups had significantly up‐regulated TIMP1 mRNA and protein compared to the si‐NC groups. ChIP also indicated that KDM1A can bind to the TIMP1 promoter region. We performed a rescue assay to confirm whether KDM1A promotes PTC cell migration and invasion by repressing TIMP1. si‐KDM1A and si‐TIMP1 co‐transfection could rescue the effects of si‐KDM1A, including the inhibition of cell migration and invasion, and partly recover MMP9 activation. The rescue assay confirmed that KDM1A depletion inhibited malignant biological behaviour by regulating TIMP1. These data indicate that KDM1A might enhance MMP9 expression and activation by inhibiting TIMP1.

Consistent with our results, some studies have shown a strong association between TIMP1 and metastasis. In pancreatic cancer cells, TIMP1 overexpression can reduce the invasive ability both in vitro and in vivo.^42^ TIMP1 can be up‐regulated by CD82 and suppresses tumour invasion in human lung carcinoma cell lines.^43^ In cervical cancer, the long non‐coding TUC338 promotes cell migration and invasion by down‐regulating TIMP1.^44^ In oral cancer, microRNA‐196 promotes invasive ability through the NME4 (NME/NM23 nucleoside diphosphate kinase 4)–JNK (JUN N‐terminal kinase)–TIMP1–MMP signalling pathway.^45^ However, TIMP1 has also been reported as an oncogene and is increased in more advanced tumours, and predicts a shorter time to relapse and worse prognosis in endometrial, breast and brain cancer.^46^, ^47^, ^48^ These discrepancies may be due to the complicated functions of TIMP1 in different cancers. TIMP1 can not only inhibit cancer by repressing MMP expression and activation, but also promotes cancer via angiogenesis, cell growth promotion and tumour inflammation.^49^ These studies all suggest the complicated role TIMP1 plays in cancer development.

Histone H3 demethylation is both positively and negatively associated with tumour metastasis. In fact, KDM1A affects tumour characteristics by regulating H3K4me2 or H3K9me2.^50^, ^51^ Here, we found that the global protein levels of H3K4me2 and H3K9me2 were up‐regulated after si‐KDM1A transfection. However, ChIP indicated that only H3K4me2 expression levels were decreased in the si‐KDM1A groups. These data demonstrate that KDM1A can repress TIMP1 expression by demethylating H3K4me2 at its promoter regions.

In conclusion, we have identified KDM1A as a metastasis promoter in PTC and that it is associated with lymph node metastasis. KDM1A enhances MMP9 expression and activation by binding to the promoter region of TIMP1 and demethylating H3K4me2, which represses TIMP1 expression. Our work uncovers a new invasive mechanism in PTC and suggests that KDM1A can be used as a potential therapeutic target in PTC (Figure 5F).

## CONFLICT OF INTEREST

Authors declare no conflicts of interest for this article.

## AUTHOR CONTRIBUTIONS

Wenqian Zhang, Wei Sun, Yuan Qin, Liang Shao and CangHao Wu performed experiments and wrote the manuscript. Liang He and Ting Zhang analysed data. Hao Zhang and Ping Zhang assisted with the design of experiments.

## References

[1] Siegel RL , Miller KD , Jemal A . Cancer Statistics, 2017. CA Cancer J Clin. 2017 ;67(1):7‐30. 28055103 10.3322/caac.21387

[2] Mao Y , Xing M . Recent incidences and differential trends of thyroid cancer in the USA. Endocr Relat Cancer. 2016;23(4):313‐322. 26917552 10.1530/ERC-15-0445 PMC4891202

[3] Mansour J , Sagiv D , Alon E , Talmi Y . Prognostic value of lymph node ratio in metastatic papillary thyroid carcinoma. J Laryngol Otol. 2018;132(1):8‐13. 29122022 10.1017/S0022215117002250

[4] Haydn T , Metzger E , Schuele R , Fulda S . Concomitant epigenetic targeting of LSD1 and HDAC synergistically induces mitochondrial apoptosis in rhabdomyosarcoma cells. Cell Death Dis. 2017;8(6):e2879. 28617441 10.1038/cddis.2017.239 PMC5520898

[5] Higgs MR , Sato K , Reynolds JJ , et al. Histone methylation by SETD1A protects nascent DNA through the nucleosome chaperone activity of FANCD2. Mol Cell. 2018;71(1):25‐41. 29937342 10.1016/j.molcel.2018.05.018 PMC6039718

[6] Wang C , Wang J , Li J , et al. KDM5A controls bone morphogenic protein 2‐induced osteogenic differentiation of bone mesenchymal stem cells during osteoporosis. Cell Death Dis. 2016;7(8):e2335. 27512956 10.1038/cddis.2016.238 PMC5108323

[7] El‐Nachef D , Oyama K , Wu YY , Freeman M , Zhang Y , MacLellan WR . Repressive histone methylation regulates cardiac myocyte cell cycle exit. J Mol Cell Cardiol. 2018;121:4933‐4944. 10.1016/j.yjmcc.2018.05.013 PMC6542357 29800554

[8] Karytinos A , Forneris F , Profumo A , et al. A novel mammalian flavin‐dependent histone demethylase. J Biol Chem. 2009;284(26):17775‐17782. 19407342 10.1074/jbc.M109.003087 PMC2719416

[9] Ismail T , Lee HK , Kim C , Kwon T , Park TJ , Lee HS . KDM1A microenvironment, its oncogenic potential, and therapeutic significance. Epigenetics Chromatin. 2018;11(1):33. 29921310 10.1186/s13072-018-0203-3 PMC6006565

[10] Chen C , Ge J , Lu Q , Ping G , Yang C , Fang X . Expression of lysine‐specific demethylase 1 in human epithelial ovarian cancer. J Ovarian Res. 2015;8:28. 25956476 10.1186/s13048-015-0155-1 PMC4429353

[11] Liu Y , Wang Y , Chen C , et al. LSD1 binds to HPV16 E7 and promotes the epithelial‐mesenchymal transition in cervical cancer by demethylating histones at the Vimentin promoter. Oncotarget. 2017;8(7):11329‐11342. 27894088 10.18632/oncotarget.13516 PMC5355268

[12] Kosumi K , Baba Y , Sakamoto A , et al. Lysine‐specific demethylase‐1 contributes to malignant behavior by regulation of invasive activity and metabolic shift in esophageal cancer. Int J Cancer. 2016;138(2):428‐439. 26240060 10.1002/ijc.29714

[13] Li W , Sun M , Zang C , et al. Upregulated long non‐coding RNA AGAP2‐AS1 represses LATS2 and KLF2 expression through interacting with EZH2 and LSD1 in non‐small‐cell lung cancer cells. Cell Death Dis. 2016;7:e2225. 27195672 10.1038/cddis.2016.126 PMC4917662

[14] Murphy G . Tissue inhibitors of metalloproteinases. Genome Biol. 2011;12(11):233. 22078297 10.1186/gb-2011-12-11-233 PMC3334591

[15] Avadanei R , Caruntu ID , Amalinei C , et al. High variability in MMP2/TIMP2 and MMP9/TIMP1 expression in secondary liver tumors. Rom J Morphol Embryol. 2013;54(3):479‐485. 24068394

[16] Venkatesan T , Alaseem A , Chinnaiyan A , et al. Overexpression modulates the angiogenesis‐related gene expression profile of prostate cancer cells. Cells‐Basel. 2018;7(5). 10.3390/cells7050041 PMC5981265 29748481

[17] Remmele W , Hildebrand U , Hienz HA , et al. Comparative histological, histochemical, immunohistochemical and biochemical studies on oestrogen receptors, lectin receptors, and Barr bodies in human breast cancer. Virchows Arch A Pathol Anat Histopathol. 1986;409(2):127‐147. 2424168 10.1007/BF00708323

[18] Annahazi A , Abraham S , Farkas K , et al. A pilot study on faecal MMP‐ 9: a new noninvasive diagnostic marker of colorectal cancer. Br J Cancer. 2016;114(7):787‐792. 26908323 10.1038/bjc.2016.31 PMC4984857

[19] Chang JW , Kang SU , Shin YS , et al. Non‐thermal atmospheric pressure plasma inhibits thyroid papillary cancer cell invasion via cytoskeletal modulation, altered MMP‐2/‐9/uPA activity. PLoS ONE. 2014;9(3):e92198. 24667444 10.1371/journal.pone.0092198 PMC3965425

[20] Shi Y , Whetstine JR . Dynamic regulation of histone lysine methylation by demethylases. Mol Cell. 2007;25(1):4933‐14. 10.1016/j.molcel.2006.12.010 17218267

[21] Amente S , Milazzo G , Sorrentino MC , et al. Lysine‐specific demethylase (LSD1/KDM1A) and MYCN cooperatively repress tumor suppressor genes in neuroblastoma. Oncotarget. 2015;6(16):14572‐14583. 26062444 10.18632/oncotarget.3990 PMC4546488

[22] Rotstein L . The role of lymphadenectomy in the management of papillary carcinoma of the thyroid. J Surg Oncol. 2009;99(4):186‐188. 19170045 10.1002/jso.21234

[23] Wada N , Masudo K , Nakayama H , et al. Clinical outcomes in older or younger patients with papillary thyroid carcinoma: impact of lymphadenopathy and patient age. Eur J Surg Oncol. 2008;34(2):202‐207. 18023321 10.1016/j.ejso.2007.10.001

[24] Gulcelik MA , Ozdemir Y , Kadri CM , Camlibel M . Alagol H. Prognostic factors determining survival in patients with node positive differentiated thyroid cancer: a retrospective cross‐sectional study. Clin Otolaryngol. 2012;37(6):460‐467. 22971040 10.1111/coa.12022

[25] Bonnet S , Hartl D , Leboulleux S , et al. Prophylactic lymph node dissection for papillary thyroid cancer less than 2 cm: implications for radioiodine treatment. J Clin Endocrinol Metab. 2009;94(4):1162‐1167. 19116234 10.1210/jc.2008-1931

[26] Meng XY , Zhang Q , Li Q , Lin S , Li J . Immunohistochemical levels of cyclo‐oxygenase‐2, matrix metalloproteinase‐9 and vascular endothelial growth factor in papillary thyroid carcinoma and their clinicopathological correlations. J Int Med Res. 2014;42(3):619‐627. 24670538 10.1177/0300060513505485

[27] Sun W , Lan X , Zhang H , et al. NEAT1_2 functions as a competing endogenous RNA to regulate ATAD2 expression by sponging microRNA‐106b‐5p in papillary thyroid cancer. Cell Death Dis. 2018;9(3):380. 29515109 10.1038/s41419-018-0418-z PMC5841310

[28] Allfrey VG , Faulkner R , Mirsky AE . Acetylation and methylation of histones and their possible role in the regulation of RNA synthesis. Proc Natl Acad Sci U S A. 1964;51:786‐794. 14172992 10.1073/pnas.51.5.786 PMC300163

[29] Forneris F , Binda C , Vanoni MA , Mattevi A , Battaglioli E . Histone demethylation catalysed by LSD1 is a flavin‐dependent oxidative process. FEBS Lett. 2005;579(10):2203‐2207. 15811342 10.1016/j.febslet.2005.03.015

[30] Burg JM , Link JE , Morgan BS , Heller FJ , Hargrove AE , McCafferty DG . KDM1 class flavin‐dependent protein lysine demethylases. Biopolymers. 2015;104(4):213‐246. 25787087 10.1002/bip.22643 PMC4747437

[31] Foster CT , Dovey OM , Lezina L , et al. Lysine‐specific demethylase 1 regulates the embryonic transcriptome and CoREST stability. Mol Cell Biol. 2010;30(20):4851‐4863. 20713442 10.1128/MCB.00521-10 PMC2950538

[32] Ding J , Zhang ZM , Xia Y , et al. LSD1‐mediated epigenetic modification contributes to proliferation and metastasis of colon cancer. Br J Cancer. 2013;109(4):994‐1003. 23900215 10.1038/bjc.2013.364 PMC3749561

[33] Lim S , Janzer A , Becker A , et al. Lysine‐specific demethylase 1 (LSD1) is highly expressed in ER‐negative breast cancers and a biomarker predicting aggressive biology. Carcinogenesis. 2010;31(3):512‐520. 20042638 10.1093/carcin/bgp324

[34] Lv T , Yuan D , Miao X , et al. Over‐expression of LSD1 promotes proliferation, migration and invasion in non‐small cell lung cancer. PLoS ONE. 2012;7(4):e35065. 22493729 10.1371/journal.pone.0035065 PMC3320866

[35] Feng J , Xu G , Liu J , et al. Phosphorylation of LSD1 at Ser112 is crucial for its function in induction of EMT and metastasis in breast cancer. Breast Cancer Res Treat. 2016;159(3):443‐456. 27572339 10.1007/s10549-016-3959-9

[36] Wang M , Liu X , Jiang G , Chen H , Guo J , Weng X . Relationship between LSD1 expression and E‐cadherin expression in prostate cancer. Int Urol Nephrol. 2015;47(3):485‐490. 25627913 10.1007/s11255-015-0915-2

[37] Kong L , Zhang G , Wang X , Zhou J , Hou S , Cui W . Immunohistochemical expression of RBP2 and LSD1 in papillary thyroid carcinoma. Rom J Morphol Embryol. 2013;54(3):499‐503. 24068396

[38] Kong LL , Man DM , Wang T , Zhang GA , Cui W . Downregulation of LSD1 suppresses the proliferation, tumorigenicity and invasion of papillary thyroid carcinoma K1 cells. Oncol Lett. 2016;11(4):2475‐2480. 27073501 10.3892/ol.2016.4244 PMC4812116

[39] Aalinkeel R , Nair BB , Reynolds JL , et al. Overexpression of MMP‐9 contributes to invasiveness of prostate cancer cell line LNCaP. Immunol Invest. 2011;40(5):447‐464. 21391788 10.3109/08820139.2011.557795

[40] Marecko I , Cvejic D , Selemetjev S , et al. Enhanced activation of matrix metalloproteinase‐9 correlates with the degree of papillary thyroid carcinoma infiltration. Croat Med J. 2014;55(2):128‐137. 24778099 10.3325/cmj.2014.55.128 PMC4009713

[41] Wang N , Jiang R , Yang JY , et al. Expression of TGF‐beta1, SNAI1 and MMP‐9 is associated with lymph node metastasis in papillary thyroid carcinoma. J Mol Histol. 2014;45(4):391‐399. 24276590 10.1007/s10735-013-9557-9

[42] Nieuwesteeg MA , Willson JA , Cepeda M , Fox MA , Damjanovski S . Functional characterization of tissue inhibitor of metalloproteinase‐1 (TIMP‐1) N‐ and C‐terminal domains during Xenopus laevis development. ScientificWorldJournal. 2014;2014:467907. 24616631 10.1155/2014/467907 PMC3925571

[43] Jee BK , Park KM , Surendran S , et al. KAI1/CD82 suppresses tumor invasion by MMP9 inactivation via TIMP1 up‐regulation in the H1299 human lung carcinoma cell line. Biochem Biophys Res Commun. 2006;342(2):655‐661. 16488391 10.1016/j.bbrc.2006.01.153

[44] Li Q , Shen F , Wang C . TUC338 promotes cell migration and invasion by targeting TIMP1 in cervical cancer. Oncol Lett. 2017;13(6):4526‐4532. 28599453 10.3892/ol.2017.5971 PMC5453013

[45] Lu YC , Chang JT , Liao CT , et al. OncomiR‐196 promotes an invasive phenotype in oral cancer through the NME4‐JNK‐TIMP1‐MMP signaling pathway. Mol Cancer. 2014;13:218. 25233933 10.1186/1476-4598-13-218 PMC4176851

[46] Honkavuori M , Talvensaari‐Mattila A , Puistola U , Turpeenniemi‐Hujanen T , Santala M . High serum TIMP‐1 is associated with adverse prognosis in endometrial carcinoma. Anticancer Res. 2008;28(5A):2715‐2719. 19035300

[47] Aaberg‐Jessen C , Christensen K , Offenberg H , et al. Low expression of tissue inhibitor of metalloproteinases‐1 (TIMP‐1) in glioblastoma predicts longer patient survival. J Neurooncol. 2009;95(1):117‐128. 19430729 10.1007/s11060-009-9910-8

[48] Dechaphunkul A , Phukaoloun M , Kanjanapradit K , et al. Prognostic significance of tissue inhibitor of metalloproteinase‐1 in breast cancer. Int. J Breast Cancer. 2012;2012:290854. 10.1155/2012/290854 PMC3440855 22988515

[49] Jackson HW , Defamie V , Waterhouse P . Khokha R. TIMPs: versatile extracellular regulators in cancer. Nat Rev Cancer. 2017;17(1):38‐53. 27932800 10.1038/nrc.2016.115

[50] Ambrosio S , Sacca CD , Amente S , Paladino S , Lania L , Majello B . Lysine‐specific demethylase LSD1 regulates autophagy in neuroblastoma through SESN2‐dependent pathway. Oncogene. 2017;36(48):6701‐6711. 28783174 10.1038/onc.2017.267 PMC5717079

[51] Cai L , Chen Q , Fang S , Lian M , Cai M . MicroRNA‐329 inhibits cell proliferation and tumor growth while facilitates apoptosis via negative regulation of KDM1A in gastric cancer. J Cell Biochem. 2018;119(4):3338‐3351. 29130516 10.1002/jcb.26497

